# Coronary Artery Disease in Male Athletes—Is Sport Healthy in the Long Run?

**DOI:** 10.3390/jcm15145329

**Published:** 2026-07-08

**Authors:** Zofia Kampka, Maciej T. Wybraniec

**Affiliations:** 1 Department of Cardiology and Structural Heart Diseases, Medical University of Silesia, 47 Ziołowa St., 40-635 Katowice, Poland; 2 Upper-Silesian Medical Center, 47 Ziołowa St., 40-635 Katowice, Poland; 3 First Department of Cardiology, School of Medicine in Katowice, Medical University of Silesia, 47 Ziołowa St., 40-635 Katowice, Poland; 4 European Reference Network on Heart Diseases—ERN GUARD-HEART, 1105 AZ Amsterdam, The Netherlands

**Keywords:** athletes, coronary artery disease, atherosclerosis, sports cardiology, coronary artery calcification

## Abstract

Physical activity wields a positive influence on atherosclerosis-related risk factors; however, the effects of high-intensity training on cardiovascular (CV) health are not unequivocal. The prevalence of coronary artery disease (CAD) in middle-aged sportsmen varies from 13.7% to 71%. Despite increased longevity and lower overall CV risk, CAD remains a serious problem in athletes, being responsible for the majority of sudden cardiac death (SCD) cases in sportsmen over 30 years. Endurance athletes engaging in high-intensity training are burdened with increased coronary artery calcification (CAC) of multifactorial pathophysiology, embracing i.a. wall shear stress, excessive reactive oxygen species, inflammatory mediators and increased levels of parathyroid hormone. Thanks to improved coronary vasodilatory capacity and coronary collateralization, sportsmen are rarely affected by hemodynamically significant coronary artery stenosis, and the atherosclerotic plaques are mostly of benign, calcified morphology. The basic screening should embrace medical history, CV risk assessment and rest electrocardiogram (ECG). Stress ECG tests, computed tomography (CT) with or without contrast, and functional imaging tests are additional diagnostic options. Because CAD in athletes remains a subject of research, the article aims at bringing closer the up-to-date findings on this matter, with a summary of diagnostic tools and clinical implications.

## 1. Introduction

Coronary artery disease (CAD) is among the major causes of cardiovascular disease-related deaths worldwide [1]. Its pathogenesis is sophisticated, including modifiable risk factors such as arterial hypertension, hyperlipidemia, diabetes mellitus, obesity, and a sedentary lifestyle, as well as non-modifiable factors including age, sex, and race [2]. Physical activity plays a role in reducing CV risk and all-cause mortality; therefore, it is advised for adults to engage in at least 75 min of vigorous exercise over 3 days or 150 min of moderate-intensity endurance exercise over 5 days per week. Increasing the amount of exercise to 150 min and 300 min per week, respectively, provides additional benefits [3]. While the role of exercise in CAD prevention is well-established, presenting a dose–effect relationship, it may seem that professional athletes, contemporary symbols of health, should be predominantly unaffected by CAD—“a disease of modern society” [1,3]. However, the existing research on the relationship between physical activity and coronary artery calcification (CAC) provides heterogeneous results, ranging from inverse, through U/J-shaped or positive dependence, to no dependence at all. The variety of outcomes may be caused by differences regarding the age of the studied population, the level of physical activity and the method of physical activity assessment [1]. Physical activity may promote coronary artery calcifications via different pathophysiological pathways, which embrace not only an excess of reactive oxygen species and inflammatory mediators, but also shear and/or hemodynamic stress affecting coronary arteries.

According to existing research, 13.7% to 71% of middle-aged endurance athletes of the male gender can be burdened with coronary plaques [1,4]. The reported prevalence of CAD varies greatly, reflecting heterogeneity in study design and participant characteristics. Factors influencing variability include differences in age, race and cardiovascular risk profiles. Moreover, the type and intensity of sport participation with cumulative training exposure were also diverse—this problem is expanded in Section 5—Exercise Type. Studies involving elderly veteran endurance athletes report higher CAD prevalence than studies evaluating a younger athletic population. Importantly, athletes constitute a heterogeneous group, and the observed burden of CAD cannot be attributed solely to exercise exposure.

The percentage of sportsmen affected by CAD is still a subject of research. However, there is no doubt that sportsmen represent a specific group of patients requiring a tailored approach. CAD is among the major causes of sudden cardiac death (SCD) in athletes aged over 30 years and is responsible for over 95% of SCD cases in athletes over the age of 40 [3,5]. It raises the need for an in-depth insight into the pathophysiology, diagnostic approach and therapy of CAD in sportsmen [5].

While typical CV risk assessment in sportsmen may lead to underestimated results, CT of coronary arteries with calcium score calculation may be useful. CAC is related to atherosclerosis and cardiovascular risk, and is a CAD marker. Increased calcium score was found to be prevalent in sportsmen with high volumes of endurance exercise training, raising the need to explain this association [6]. This narrative review was based on a comprehensive literature search conducted in PubMed, Scopus, and Web of Science. Publications related to coronary artery disease in athletes were identified using combinations of the following keywords: “coronary artery disease”, “atherosclerosis”, “coronary artery calcification”, “athletes”, “endurance athletes”, “exercise”, “high-intensity training”, “sports cardiology”, “sportsmen, and “sudden cardiac death”. Priority was given to original studies, meta-analyses, systematic reviews, and current clinical guidelines published in English. Additional relevant articles were identified through manual screening of reference lists.

This article aims at providing a comprehensive overview of CAD in athletes by summarizing evidence regarding the prevalence, pathophysiological mechanisms, and clinical characteristics of CAD in this population. It also discusses available diagnostic and screening strategies and highlights the clinical implications for cardiovascular risk assessment, prevention, and management.

## 2. Pathophysiology

**Figure 1:**
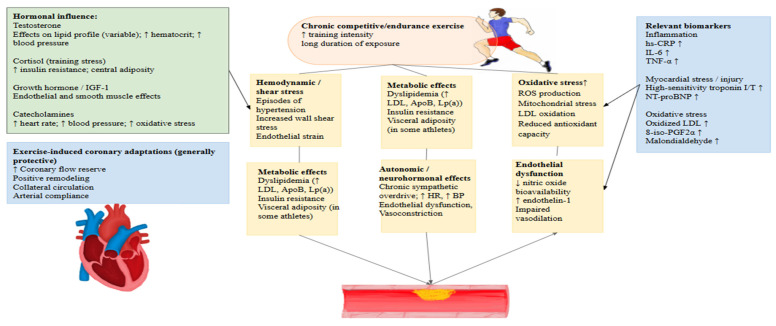
Potential underlying mechanisms of coronary artery disease (CAD) in sportsmen [1,6].

The relationship between high-intensity exercise and CAC and its underlying mechanisms is not well-established. Presumably, the pathophysiology is multifactorial and complex. Potential causes embrace wall shear stress, increased levels of parathyroid hormone and testosterone, and excessive reactive oxygen species and inflammatory mediators [Figure 1]. Shear stress affects the atherosclerotic process by modulating the gene expression of vascular endothelial cells and altering their function [7]. The concentration of endothelin-1 is significantly higher in athletes compared with the general population, which can be the result of oxidative stress. Higher endothelin-1 concentration translates into impaired vasodilation and increased vasoconstriction, potentially leading to coronary microvascular dysfunction [8].

While the influence of sole hemodynamic stress, including epicardial coronary arteries flexing during vigorous heart contractions and high blood pressure on exertion, is poorly studied on healthy vessels in athletes, it is known for its ability to accelerate atherosclerosis in sportsmen with pre-existing coronary artery disease [6,9]. However, the existing research includes a relatively small number of participants [10].

Transient, yet repeated, exposure to increased parathyroid hormone (PTH) levels can translate into the progression of vascular and valvular calcification, similarly to vitamin D and magnesium deficiency, which can be found in the athletic population [1,6]. The presumed causes of PTH rise involve a decrease in ionized calcium concentration on exertion, catecholamine secretion and acidosis [11]. Elevated PTH, calcium and phosphate concentrations are predictive factors of arterial calcification, forming a potential link between exercise and CAC [12].

While physiologic testosterone concentrations do not seem to negatively affect cardiovascular health, anabolic–androgenic steroid abuse results in plaque build-up, which can lead to myocardial ischemia [13,14]. The role of testosterone is further complicated by contradictory research results on its influence on atherosclerotic calcification. However, there is no doubt that CAC is predominantly prevalent in male athletes [6]. The protective role of sex hormones in women is based on their impact on lipid and glucose metabolism and blood pressure, thereby lowering the risk of CAD. The role of gender hormones is sophisticated, as both testosterone deficiency and excess are potentially harmful [15]. Testosterone treatment translates to atherosclerosis progression and may negatively influence arterial remodeling. However, the exact mechanism of the atherogenic effect is not clear, and it does not seem to be mediated by changes in the plasma lipoprotein profile. Testosterone may directly influence arterial intima by plasma LDL metabolism, leading to increased arterial cholesterol accumulation and atherosclerosis progression. It was suspected that the atherogenic effect of androgens was mediated by impaired endothelial function and endothelium-dependent vasomotor response; however, this hypothesis has not been proved [16].

Moreover, it seems that the burden caused by prior traditional risk factors, such as tobacco use, hypertension and hyperlipidemia, is not erased, even when one decides to increase sport activity [17].

## 3. Coronary Atherosclerosis Assessment

Assessing the risk factors for CAD and looking for its symptoms is vital in the athletic population. The first-line diagnostic options include electrocardiogram, echocardiogram and cardiovascular risk assessment. However, these standard methods are characterized by limited sensitivity, necessitating the use of other diagnostic tools [6].

### 3.1. Resting and Exercise Electrocardiogram

The ECG criteria from the European Society of Cardiology (ESC-2005), which used to be recommended for use in young athletes, can be applied to athletes >35 years in order to detect high-risk cardiovascular conditions, with CAD being the most common finding, encompassing pathologic Q-waves, ST-segment deviation, T-wave inversion and left bundle branch block. However, there is a limited body of evidence regarding the predictive value of ECG findings in master athletes, further complicated by the need to differentiate between physiologic cardiac adaptation and pathological findings, with the border being frequently blurred [18]. Moreover, while the ESC-2005 criteria are more sensitive in detecting high-risk cardiovascular conditions than the Seattle criteria, their false positive rates are also higher [19]. Taking into account all of the above, a resting ECG does not make a perfect screening tool for CAD detection. However, it is still superior in sensitivity compared to medical history and physical examination in CV screening [18,19]. These dilemmas raise the need to use imaging techniques [19].

There are no existing official guidelines on the management of asymptomatic athletes with CAC. The ESC Guidelines on Sports Cardiology and Exercise in Patients with Cardiovascular Disease from 2020 advise screening for CAD in master athletes >35 years of age, based on symptoms and maximal exercise test results. However, sportsmen with mild to moderate atherosclerotic plaques may remain unidentified. Moreover, the evidence for CV screening in this group is limited. CV screening in young athletes may translate into decreasing CV morbidity and includes medical history, physical examination and ECG. The presence of symptoms or abnormal findings on screening tests requires further investigation [3].

Additional tests are reliant on local availability, costs and logistics. The usefulness of the traditional ECG-treadmill test is questionable. On one hand, athletes have high vasodilatory capacity and coronary flow reserve; therefore, the test’s sensitivity and specificity are limited, especially in the detection of subclinical coronary artery disease [20,21]. On the other hand, according to official recommendations, exercise ECG is reserved for asymptomatic sportsmen who are at a high risk of CAD, according to SCORE2 and SCORE2-OP algorithms. The SEEPRED study advocates the use of exercise ECG in sportsmen over 35 years with at least two CVD risk factors. The most significant risk factors involve male sex, age (≥60), concomitant diseases (dyslipidemia and hypertension), family history of SCD or CVD, and high CVD risk based on algorithms (SCORE2 > 7.5% in patients < 50 years old and >10% between 50 and 69 years; SCORE2-OP > 15% in patients > 70 years). Being burdened with at least two CVD risk factors is among the best predictors for the presence of significant CAD. While the value of exercise ECG in detecting CAD in low to intermediate CV risk athletes is limited, its use in high-risk sportsmen, according to the SEEPRED study, is characterized by high negative predictive value (>99%) with specificity and sensitivity of 93% and 77% respectively [22].

Performing exercise ECG may be considered to detect inducible ischemia, especially in athletes with a calcium score ≥400 Agatston units (AU) and/or luminal stenosis >50% [1]. However, it seems that a conventional exercise ECG protocol in athletes may prove insufficient to trigger cardiac symptoms. It is suggested not to terminate the stress ECG test when reaching a predefined target heart rate but to continue the test until the maximal possible exercise capacity is reached. This heightens the chance of symptom detection. This explains why athletes may need individualized testing, embracing alterations of exercise duration and/or intensity. The accessibility of individualized testing remains low [23].

### 3.2. Computed Tomography

Additional testing embraces computed tomography (CT) with or without contrast. Non-contrast CT enables the calculation of a calcium score, which translates to coronary atherosclerotic burden and cardiovascular disease occurrence. The calcium score takes into consideration the total CAC area and its density, which is based on the highest Hounsfield units. Interestingly, there is an inverse correlation between the density and CV risk [3]. There is no evidence supporting calcium score assessment in athletes based solely on their exertion history. Asymptomatic sportsmen with a 1-year (atherosclerotic cardiovascular disease (ASCVD) risk between 5 and 20%, aged 40–75 should be the target group [1]. According to the MARC study, which embraced both professional and leisure asymptomatic sportsmen aged 45 years and more, who had no abnormalities in sports medical examination (SME—embracing i.a. bicycle exercise ECG), even one in every five participants was affected with relevant CAD detected by CT. However, whether the use of CT in additional testing reduces the incidence of CV exercise-related events in older athletes still requires further research [24].

CT with contrast (coronary CT angiography) not only allows for the determination of the coronary artery lumen but also the morphology of the plaque, dividing plaques into calcified (“chalk”), non-calcified (“cheese”), and mixed. This division enables in-depth CV risk assessment, with “chalk” plaques being of the lowest risk for a CV event. Moreover, “cheese” plaques may not be detected in non-contrast CT due to low density [3]. CT angiography is advised for low- to intermediate-risk recreational endurance athletes for the detection of occult coronary artery disease [25]. It seems that even asymptomatic professional athletes, who are at low to intermediate CAD risk, may benefit from CT angiography with FFR-CT (fractional flow reserve), which aims at identifying occult coronary artery disease and improves the accuracy of CT angiography. FFR-CT also allows for the reduction in the number of patients referred for redundant invasive coronary angiography [20]. CT angiography also plays an important role in the identification and significance assessment of anomalous coronary arteries, which may be the cause of myocardial infarction in the young population [17]. Functional evaluation on exertion is required to assess the clinical significance of these findings [26].

Because of certain limitations of CT scans concerning spatial resolution, such novel diagnostic tools as micro-CT and photon-counting detectors may be useful in detecting early stages of atherosclerotic calcifications and their progression; however, their clinical application is not yet adopted [27].

### 3.3. Cardiac Magnetic Resonance

While the role of cardiac magnetic resonance imaging (CMR) in coronary artery disease in sportsmen is limited, it plays a significant role in differentiating between physiologic hypertrophy, which is a result of sport adaptation, and pathological hypertrophy, which suggests the presence of hypertrophic cardiomyopathy [17]. A relationship between calcium score and the number of marathons run and late gadolinium enhancement (LGE) on CMR has been found, which can reveal subclinical myocardial damage [25]. LGE is an indicator of subendocardial fibrosis, presumably a result of subclinical myocardial infarction (MI) caused by coronary spasm, demand ischemia, and micro-emboli. The presence of LGE accompanied by CAC is associated with an increased risk of adverse outcomes [9]. However, the clinical implications of these findings have not yet been established. CMR may be found useful in the future to detect ischemia in asymptomatic athletes with a negative stress ECG test result [25].

### 3.4. Stress/Exercise Tests

Stress echocardiography is helpful in detecting exercise-induced ischemia, either caused by CAD or anomalous coronary arteries, in asymptomatic athletes, often with abnormalities on rest ECG. An exercise test performed on a bed-cycle ergometer is the preferred option in athletes over pharmacologically induced stress. An exercise echocardiography test is a non-invasive and easily accessible diagnostic option; however, it requires an experienced echocardiographer due to the short time of image acquisition [26].

Cardiorespiratory exercise testing (CET), despite not being commonly used, provides information on ventilatory parameters, respiratory oxygen uptake and carbon dioxide production. CET is performed in order to evaluate unexplained symptoms such as exertional breathlessness or fatigue. It can also serve to establish the appropriate level of training intensity in athletes and patients with CV disease [26]. Exercise testing of cardiorespiratory fitness can be useful in CV risk stratification in athletes, with a higher baseline fitness level being associated with lower CV risk, regardless of CAC presence [6]. The lack of reference values for CET in athletes, however, limits its wider use for the time being [26].

Stress CMR may be applicable in excluding significant stenosis with a high negative predictive value; however, its role in asymptomatic athletes has not yet been established. Moreover, the cost-effectiveness ratio of stress CMR has not yet been evaluated [17]. The results of the EMPIRE trial, which examined the diagnostic value of exercise stress CMR in patients of intermediate CV risk, were promising—they showed no inferiority in terms of diagnostic accuracy to FFR. Stress CMR using a bed-cycle ergometer may become one of the preferred diagnostic options in the future; however, it still requires multi-center studies in varying cohorts [27].

### 3.5. Other Noninvasive Tests

Myocardial perfusion scintigraphy (MPS) embraces positron emission tomography (PET) and single-photon emission computed tomography (SPECT) and belongs to modern imaging techniques that enable further diagnostic evaluation of sportsmen with suspected CAD. Because of its high accuracy in CAD detection, it is useful in patients with a positive stress ECG result. The specificity of SPECT values in athletes can be reduced due to coexisting LV hypertrophy, though. Myocardial perfusion scintigraphy belongs to additional tests for more accurate evaluation of asymptomatic athletes >35 years with CV risk factors. Being now a complementary method to stress ECG and CT, it is possible that it will play a greater role in the future [26]. Nevertheless, a study by van de Sande et al. stands in contrast to the existing literature. This research aimed at evaluating the prognostic value of MPS in significant CAD detection in asymptomatic athletes with abnormal stress ECG results and concluded that the positive predictive value of MPS in this group was as low as 20%. These results may be explained by endothelial dysfunction hindering myocardial perfusion or atherosclerosis located in the microvasculature. It seems that MPS can lead to unnecessary procedures such as coronary angiography; therefore, other diagnostic options should be implemented instead [28].

Myocardial blood flow and myocardial flow reserve, in order to detect abnormalities in microvasculature, can be assessed with Rubidium-82 (82Rb) PET and perfusion CMR. However, their availability is often limited, and the literature on using them in athletes is scarce [28].

Carotid ultrasound, relying on the measurement of the intima–media complex, can be one of the diagnostic tools allowing for improvement of CV risk estimation. In a study by Burgstahler et al., which included 49 asymptomatic male marathon runners older than 45 years, the carotid ultrasound’s positive predictive value was 70.6%, and its negative predictive value was 67.7% for coronary atherosclerosis prediction. Although the accuracy of carotid ultrasound is lower than that of a non-contrast CT, this diagnostic tool is widely available, relatively cheap and does not expose the patient to radiation. While its diagnostic value seems to be higher than that of resting and stress ECG, more research is needed for an in-depth evaluation of this method in sportsmen [29].

Pulse wave velocity (PWV), as a marker of arterial stiffness, has limited clinical significance. It may be of limited additional value in asymptomatic, middle-aged sportsmen for identifying subclinical CAD. It can contribute to CV risk prediction and mortality in younger athletes at intermediate risk of CAD, where traditional risk assessment can be underestimated [30].

### 3.6. Invasive Evaluation

Further steps are dependent on the results of prior tests and include invasive coronary angiography with complementary diagnostic methods, such as intravascular imaging [1]. Optical coherence tomography (OCT) may be especially useful in diagnosing plaque erosion, which is more often caused by physical activity than plaque rupture [31]. Moreover, it helps to identify high-risk plaques characterized by a fissured or thin cap, so-called thin-cap fibroatheroma (TCFA, fibrous cap thickness <65 μm), endothelial denudation with superficial platelet aggregation, and a large lipid core (comprising >40% of the plaque’s volume). These high-risk plaques are especially vulnerable to shear stress, which is present in athletes [4]. On the other hand, atherosclerotic plaques in athletes are predominantly calcified; therefore, their likelihood of rupture is lower [1].

## 4. Unique Features of Atherosclerosis in Athletes

**Figure 2:**
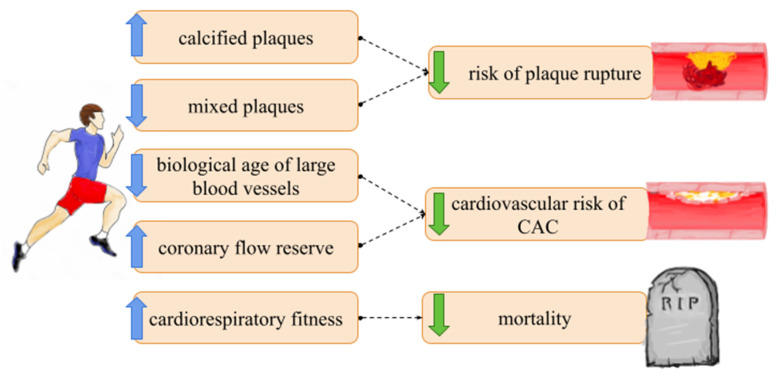
Specific features of cardiovascular health in sportsmen [1,20]. CAC—coronary artery calcification.

The specific features of cardiovascular health and atherosclerosis in sportsmen are presented in Figure 2. While a higher calcium score in athletes is similarly associated with increased CV risk as in the general population, the overall atherosclerotic burden may be overestimated in athletes [1,21]. Undoubtedly, physical exertion favors sportsmen with increased longevity. Despite a higher burden of atherosclerotic plaques in athletes, their “chalk” morphology results in a lower rupture likelihood, thus resembling the calcification process during statin therapy [1]. Higher levels of high-density lipoproteins (HDL) present in recreational endurance athletes engaging in moderate- and high-intensity training may be one of the underlying causes, translating into lipid-plaque interactions [21].

While calcified plaques, with their stable character, do not increase the risk of MI through the mechanism of plaque rupture, they can contribute to a mismatch between oxygen supply and demand due to significant coronary stenosis, causing type II MI. This can further lead to myocardial scarring and fatal arrhythmias. Type II MI is the most common underlying cause of sudden cardiac arrest (SCA) in elderly endurance athletes [32].

On the other hand, athletes have a lower prevalence of high-grade stenosis (>50%) compared to their sedentary controls. Due to physical adaptation, they are characterized by improved coronary vasodilatory capacity and coronary collateralization [20]. In a study by Feuchtner et al., which included recreational endurance athletes, there was a high prevalence of subclinical CAD in sportsmen engaging in regular exercise for over a year, training ≥3 times per week for at least 1 h [21]. This stresses the need for a personalized approach and management in professional sportsmen.

Interestingly, in relation to athletes, the term “the risk paradox” was coined. While long-term CV risk is lowered by physical exercise, it is simultaneously temporarily heightened during and after exertion, especially in sportsmen with unknown cardiac disease [24]. The underlying causes include transient prothrombotic activity and an increase in inflammatory factors, which destabilize atherosclerotic plaque and, as a result, can lead to reduced coronary perfusion and ischemia. Other vital peri-exertion factors are increased sympathetic activity and reduced vagal stimulation, heightened catecholamine levels and electrolytes imbalance. Post-exertion CV risk is augmented by dehydration, arterial vasodilation, reduced venous return and cardiac output, especially when triggered by abrupt exercise cessation. Reduced blood pressure causes a worsening of coronary perfusion [5,31]. Endurance training results in a transient troponin and brain natriuretic peptide (BNP) rise, hindered myocardial relaxation, and right and left ventricular systolic dysfunction. The rise in biomarkers of myocardial damage is of unknown clinical significance. Myocyte necrosis may translate into heightened serum troponin levels and range from subtle myocardial inflammation to microinfarction. Repeated events can eventually lead to arrhythmia and adverse cardiac remodeling. A rise in BNP levels can be a result of prolonged and repeated wall stress caused by increased cardiac output, revealing right ventricular dysfunction [9].

Atherosclerosis belongs to major exercise-related SCDs in the athletic population over 30 years of age; however, young athletes are not free from trouble [3]. Admittedly, the occurrence of MI in young athletes is not common, yet in males it is among the leading causes of SCD. MI in young athletes is rarely atherosclerosis-related, and there is a wide range of underlying causes, such as coronary artery pathology (SCAD—spontaneous coronary artery dissection, coronary artery constriction, compression of the artery, anomalous anatomy), hypercoagulability, left ventricular hypertrophy, androgenic-anabolic steroid use and plaque erosion [31]. Coronary artery anomalies are responsible for over 10% of SCD in young athletes, with the most common and serious one being the left main coronary artery originating from the right sinus of Valsalva with its course between the aorta and pulmonary trunk. CAD-related SCD in young athletes affects predominantly those aged 30–35 and is caused by left anterior descending artery obstruction [33]. The survival rate of SCD in athletes is very low, stressing the need for early screening for CAD in asymptomatic patients [31].

## 5. Exercise Type

According to novel research by Aengevaeren VL et al., it is predominantly exercise intensity, rather than exercise volume, that plays a role in CAD progression. Higher exercise volume in middle-aged men during a 6-year follow-up was not associated with an increase in calcium score or plaque progression. However, very vigorous exercise, defined as ≥9 metabolic equivalents of task (METs), resulted in coronary atherosclerosis progression (mostly calcified plaques) and a rise in calcium score. No such association was found in sportsmen engaging in less vigorous (6 to 9 METs) exercise [34].

While exercise type is closely related to exercise intensity, the influence of particular sport disciplines on CAD occurrence and progression is still a subject of research. The results of the MARC study revealed a lower prevalence of CAC and CAD in cyclists compared to other sportsmen, i.e., runners. The potential underlying mechanisms were concluded as lower exercise intensity and lower bone mineral density in cyclists, influencing parathyroid hormone levels [35]. Swimming, aerobics and racquet sports were associated with a significant reduction in CVD mortality; however, this cohort research included the general population rather than professional sportsmen [36]. Table 1 presents a summary of existing research on CAD prevalence in athletes and the influence of competitive physical activity on CV health.

Taking into consideration the fact that many athletes engage in more than one type of sport and the limited research on exercise type and CAD, the discipline should not be a factor influencing clinical decisions.

## 6. Hyperlipidemia in Sportsmen

Dyslipidemia is one of the vital and well-known risk factors for CAD and often remains underdiagnosed. Genetic factors play a pivotal role; however, dietary fat, sugar and alcohol intake, smoking, physical activity, body mass index and gender hormones are not negligible [45].

Dietary modification and physical activity, apart from pharmacotherapy, are the basic interventions in hyperlipidemia treatment. A meta-analysis by Smart NA et al. included 8673 individuals (5273 exercising participants and 3400 sedentary controls), with the exercising group engaging in predominantly vigorous or moderate-intensity training 3 times per week. Three weekly sessions of vigorous or high-intensity aerobic or combined training (aerobic and resistance training) result in significant total cholesterol (TC), low-density lipoproteins (LDL), very low-density lipoproteins (VLDL) and triglycerides (TG) reduction. All types of training (aerobic, resistance, combined) increase HDL levels. Why resistance training significantly affects only the HDL level is still a subject of research [32]. Lipoprotein(a)—Lp(a), one of LDL subfractions, belongs to acknowledged risk factors for atherosclerosis; however, it does not differ significantly among endurance athletes, power athletes and sedentary controls and is genetically determined [33,46]. Lp(a) concentration can be transiently elevated after endurance exercises due to systemic and muscular stress reaction. Moreover, in power athletes, Lp(a), as well as HDL, may be negatively affected by anabolic–androgenic steroid use (AAS) [33]. Importantly, increased Lp(a) is associated with a lower threshold for provoked coronary vasospasm, which can result in myocardial infarction, ventricular arrhythmias and sudden cardiac death [47].

Although endurance athletes are favored with lower TG concentration and improved HDL to LDL ratio, dyslipidemia belongs to the most common CV risk factors in the general athletic population [33,48]. Elderly males engaging in skill sports, with a higher fat mass percentage, are especially at risk. Therefore, athletes should be actively screened for hyperlipidemia and require effective management [48]. It is suggested to make clinical decisions based on calcium score and estimated 10-year CVD risk. A calcium score > 100 AU, meaning at least moderate risk, or estimated 10-year CVD risk exceeding 10% necessitate implementation of statin therapy. The therapeutic aims are no different than those for the general population [23]. Lipid-lowering pharmacotherapy with statins may translate into higher myopathy prevalence in endurance sportsmen [49]. Moreover, statin use, regardless of its type, heightens the risk of tendinopathy, including hand, wrist, elbow, shoulder, and Achilles, especially at the beginning of the therapy [50]. Therefore, lipid-lowering pharmacotherapy in active sportsmen should always be implemented with close monitoring of possible side effects, but it should not be delayed. Due to insufficient data on the athletic population, the use of ezetimibe and PSK9-inhibitors should be based on recommendations for the general population [23].

## 7. Clinical Management

### 7.1. Risk Assessment

Increased risk of SCD and non-fatal cardiac events in athletes necessitates the pre-participation screening process. The most basic examination embraces taking the medical history with SCORE2/SCORE2-OP algorithms assessment, rest ECG and—in some cases—stress ECG test [3,28]. The goal is to detect silent CAD in athletes above 35 years of age, especially in terms of pre-participation screening [51]. Middle-aged patients who engage in endurance leisure activities, especially after a long period of sedentary lifestyle, should also be considered for prescreening. Patients taking part in amateur marathons or ultramarathons, rowing or competitive cycling ought to be informed about the sport-related risk caused by high-intensity training [52].

Gathering the medical history embraces data on CAD symptom presence, family history (MI, SCD, CAD risk factors and genetic diseases running in the family), as well as CV risk factors [51]. Athletes are affected by both traditional and “non-traditional” CV risk factors. While the prevalence of the traditional ones, such as diabetes, hypertension, dyslipidemia and tobacco use, is limited, it is not without significance. “Non-traditional” CV risk factors embrace poor dietary patterns, sleep deprivation and exposure to psychosocial stress [49]. The influence of legal performance-enhancing substances such as androgen prohormones, caffeine and creatine on CV health requires further examination due to mixed findings in research studies [53].

### 7.2. Diagnostic Pathway

Sedentary individuals and athletes at high CV risk engaging in high-intensity training should undergo a stress ECG test [3]. According to research by van de Sande et al., asymptomatic athletes with abnormal stress ECG test results and myocardial perfusion scintigraphy results indicative of myocardial ischemia but without obstructive CAD were burdened with coronary microvascular dysfunction. The significance of this finding remains unclear. Although the study group was small (9 sportsmen), the research raises concern about the potentially important clinical implications and stresses the need for further research [8].

The negative result of an ECG stress test does not exclude mild or moderate obstructive CAD. Therefore, it seems reasonable to perform additional testing in asymptomatic athletes >35 years old with high or very high CV risk [3]. The diagnostic process can incorporate CT angiography or a functional imaging test, with stress echocardiogram and stress SPECT being of choice due to their noninvasive character and availability [3,4]. Asymptomatic athletes with CAC should be counseled on further sport activity, stressing the probable risk of high-intensity training, as well as potentially alarming symptoms necessitating an urgent doctor’s appointment. Syncope, palpitations, exertional chest pain, dyspnea, fatigue or sudden sports results worsening should be perceived as red flags [1,6].

### 7.3. Pharmacotherapy

#### Symptomatic Athletes

Symptomatic sportsmen should be treated according to the guidelines for the general population. Athletes diagnosed with CAD during coronary angiography, with inducible ischemia on a stress ECG test, or with a calcium score > 100 AU should be evaluated in terms of LV function. Moreover, atherosclerotic CAD demands modification of risk factors and implementation of preventive treatment [3]. Whether sportsmen with high-risk plaque prevalence should be managed more carefully is a question of debate due to its complexity. It is advised to thoroughly assess the risk with functional imaging tests or CT angiography [54]. Because of temporary CV risk heightening during and after exertion, it seems reasonable to modify the training regime towards moderate or low intensity alongside with intensification of pharmacological therapy to reach the LDL target level. This requires clinician–athlete dialogue to discuss limiting participation in professional competitions and high-intensity training and turning to low-intensity competitive sports instead [20].

In athletes undergoing coronary angiography, a complete revascularization strategy is chosen due to the heightened risk of atherosclerosis-related SCD. Dual antiplatelet therapy after revascularization carries an increased risk of bleeding, which can be especially concerning in athletes engaging in high-impact and contact sports. It should be implemented in accordance with contemporary guidelines and requires advising the patient on minimizing the risk of bleeding by avoiding high-risk sports [23].

### 7.4. Specific Cases—Anomalous Coronary Anatomy, Vasospastic Angina, Coronary Thromboembolism

Patients diagnosed with anomalous coronary anatomy, such as a wrong sinus origin with the coronary artery passing between the pulmonary trunk and aorta, should refrain from competitive sports [55]. In some cases, surgical correction is indicated, allowing the athlete to return to sport [4]. Asymptomatic myocardial bridging is not a contraindication to limiting physical activity. In the case of ischemic symptoms’ occurrence, athletes should be counseled on switching to low-intensity training [54].

Vasospastic angina belongs to the rare causes of chest pain in sportsmen, with only a few cases reported in the literature, and is probably a result of coronary smooth muscle hyperreactivity and endothelial dysfunction, with genetic factors also being involved. It can potentially lead to myocardial infarction, ventricular arrhythmias and sudden cardiac death. While it is typical for vasospastic angina to appear at rest between the night and early morning, it may also occur on exertion, resembling typical angina. An episode of vasospastic angina may be triggered by morning exertion and be absent during afternoon training. The management of sportsmen is no different than in the general population [47,55].

One of the rare yet significant and often underdiagnosed conditions is coronary thromboembolism arising from aortic atherosclerosis. Commonly serving as a prognostic marker for CV disease, aortic atherosclerosis can rarely cause thromboembolic events and aortic complications, such as dissection or pseudoaneurysm. Coronary thromboembolism is one of the potential MINOCA pathomechanisms, and management of risk factors is the cornerstone of primary prevention [56].

## 8. Future Perspectives

A lot of important questions on this topic remain unanswered. Current evidence is limited due to the observational character of most studies, their relatively small sample size and scarce long-term outcome data. It remains uncertain whether increased CAC in athletes represents a benign adaptation to lifelong exercise or should be treated as a marker of accelerated atherosclerosis. More research is needed to determine whether CAC in athletes has the same prognostic strength as CAC in sedentary people and what the optimal screening strategies and management of asymptomatic athletes with CAC are. Developing evidence-based management guidelines is crucial, as currently no specific guidelines are established for many clinical scenarios involving athletes. Future studies should concentrate on improving risk stratification and provide long-term observations from large prospective cohort studies.

## 9. Conclusions

Physical activity remains beneficial for CV health; however, the relationship between exercise and CAD is more complex than the “exercise is always protective” paradigm. High-intensity endurance training seems to be associated with a greater prevalence of CAC, but these plaques are more stable in comparison to those in sedentary individuals. This creates the “athlete’s paradox”—despite a higher CAC burden, overall CV mortality is lower. Still, CAD remains the major cause of SCD in athletes over 30 years old, being responsible for >95% of SCD cases in athletes over 40. Screening for CAD should be individualized and based on age, symptoms, family history, CV risk profile and planned high-intensity competition. Because of good exercise capacity and physiological cardiac adaptations in athletes, standard screening methods (medical history, resting ECG, exercise ECG) may be insufficient. Imaging tests, such as coronary CT angiography, should be considered particularly in athletes >35 years with intermediate or high cardiovascular risk, abnormal results from stress testing and suspicious symptoms. Traditional cardiovascular risk factors (hypertension, dyslipidemia, smoking, diabetes) continue to matter even in highly trained athletes. Although athletes often have higher HDL and lower triglycerides, dyslipidemia remains common and should be treated the same as in the general population. This article summarizes the problem of CAD in athletes, emphasizing practical clinical knowledge on screening, diagnosing and management, concluding that a long run does not always pay off!

## Figures and Tables

**Table 1:** The influence of competitive physical activity on cardiovascular health—coronary artery disease prevalence, significance and progression.

Study	Number of Participants	Sports Discipline	Endpoint	Results
Braber et al. 2016 [24] Occult coronary artery disease in middle-aged sportsmen with a low cardiovascular risk score: The Measuring Athlete’s Risk of Cardiovascular Events (MARC) study	318	different disciplines (i.a. cycling, long distance running, fitness)	occult CAD prevalence with non-invasive diagnostic methods	60 participants (18.9%) were diagnosed with occult CAD
Möhlenkamp et al. 2008 [25] Running: the risk of coronary events: Prevalence and prognostic relevance of coronary atherosclerosis in marathon runners	1188 (108 athletes)	marathon running	prevalence of CAC and coronary events with non-invasive diagnostic methods	Higher rate of calcium score ≥ 100 AU in marathon runners than in age and risk factor-matched controls 4 runners (3.7%) experienced coronary events
Tsiflikas et al. 2015 [37] Prevalence of Subclinical Coronary Artery Disease in Middle-Aged, Male Marathon Runners Detected by Cardiac CT	50	marathon running	prevalence of CAD with non-invasive diagnostic methods	24 participants (48.0%) were diagnosed with CAD, including 1 case (2.0%) of significant CAD
Merghani et al. 2017 [38] Prevalence of Subclinical Coronary Artery Disease in Masters Endurance Athletes With a Low Atherosclerotic Risk Profile	152	long-distance running and cycling	prevalence of CAD with non-invasive diagnostic methods	Male athletes are more likely to have a calcium score > 300 AU/coronary plaques compared with sedentary males with a similar risk profile
Aengevaeren et al. 2017 [39] Relationship Between Lifelong Exercise Volume and Coronary Atherosclerosis in Athletes	284	different disciplines (i.a. cycling, running, athletics, football)	prevalence of CAD with non-invasive diagnostic methods	Participants with high PA had a higher prevalence of CAC and atherosclerotic plaques; however, the composition of plaques was more benign
De Bosscher et al. 2023 [40] Lifelong endurance exercise and its relation with coronary atherosclerosis	558 (382 athletes)	cycling, running, triathlon	prevalence of CAD with non-invasive diagnostic methods	Athletes had more coronary plaques, including more non-calcified plaques in proximal segments, than fit and healthy individuals with a similarly low cardiovascular risk profile
Feuchtner et al. 2020 [20] Differences in coronary vasodilatory capacity and atherosclerosis in endurance athletes using coronary CTA and computational fluid dynamics (CFD): Comparison with a sedentary lifestyle.	100 (52 athletes)	running, cycling, alpine mountain endurance sports, swimming, dancing	prevalence of CAD with non-invasive diagnostic methods	Endurance training improves coronary vasodilatory capacity and reduces high-risk plaque and mixed non-calcified plaque burden
Kim et al. 2020 [41] Exercise-induced hypertension can increase the prevalence of coronary artery plaque among middle-aged male marathon runners.	50	marathon running	prevalence of CAD in athletes with EIH with non-invasive diagnostic methods	The prevalence of coronary artery plaque was higher in marathon runners with EIH than in marathon runners with normal BP (12 (42.9%) vs. 1 (4.5%) respectively)
Roberts et al. 2017 [42] Fifty Men, 3510 Marathons, Cardiac Risk Factors, and Coronary Artery Calcium Scores.	50	marathon running	CAC prevalence with non-invasive diagnostic methods	Calcium score is related to CAD risk factors and not the number of marathons run or years of running
Guy et al. 2019 [43] Incidence of major adverse cardiac events in men wishing to continue competitive sport following percutaneous coronary intervention.	108	different disciplines (i.a. cycling, marathon running, mountain biking)	Incidence of major adverse cardiac events (MACE) in men with CAD who perform intensive physical activity after a stenting procedure	MACE occurred in 17 patients (15.7%) during the follow-up period (57.6 ± 46.0 months) 4.6% of participants had isolated stent restenosis, and 3.7% each had stent restenosis plus new coronary stenosis, stent thrombosis or isolated new coronary stenosis
S Fyyaz et al. 2024 [44] Longitudinal insights into coronary plaque amongst master athletes	96 (72 athletes)	long-distance running	CAD progression in athletes compared with sedentary controls, using non-invasive diagnostic methods	No significant differences were detected in CAD progression in athletes compared with sedentary controls; however, exercise induces plaque stabilizing effect

AU—Agatston units, BP—blood pressure, CA—cardiac arrest, CAC—coronary artery calcification, CAD—coronary artery disease, CCTA—coronary CT angiography, CT—computed tomography, cTnI—cardiac troponin I, CV—cardiovascular, CVD—cardiovascular disease, EIH—exercise-induced hypertension, LAD—left anterior descending artery, MACE—major adverse cardiovascular events, MI—myocardial infarction, PA—physical activity, SCD—sudden cardiac death.

## Data Availability

No new data were created or analyzed in this study.
